# Mitochondrial creatine kinase 1 regulates the cell cycle in non-small cell lung cancer via activation of cyclin-dependent kinase 4

**DOI:** 10.1186/s12931-023-02417-2

**Published:** 2023-04-15

**Authors:** Mengjie Yang, Xuecen Wang, Zhihua Ye, Tingyu Liu, Yuan Meng, Youfa Duan, Xuexia Yuan, Xin Yue, Wenbin Deng, Ran-yi Liu

**Affiliations:** 1 School of Pharmaceutical Sciences (Shenzhen), Shenzhen Campus of Sun Yat-Sen University, Shenzhen, China; 2 State Key Laboratory of Oncology in South China, Collaborative Innovation Center for Cancer Medicine, Sun Yat-Sen University Cancer Center, Guangzhou, China; 3 Department of Radiation Oncology, The First Affiliated Hospital, Sun Yat-Sen University, Guangzhou, China; 4 Department of Medical Oncology Center, Zhongshan People’s Hospital, Zhongshan, Guangdong Province China; 5 The Second Affiliated Hospital, Guangzhou Medical University, Guangzhou, China

**Keywords:** CKMT1, NSCLC, Cell cycle, CDK4, Paclitaxel

## Abstract

**Background:**

Non-small cell lung cancer (NSCLC) is the main type of the most common malignant tumor in the world. Previous studies have shown that the expression level of mitochondrial creatine kinase 1 (CKMT1) is abnormal in NSCLC, but the mechanism of its effect remains unclear. Therefore, in this study, we intend to clarify the potential mechanism of CKMT1 in NSCLC and provide the theoretical basis for the clinical application of CKMT1.

**Methods:**

The function of CKMT1 in NSCLC was identified by analyzing the GEO dataset and evaluating using in vitro and in vivo models. Protein mass spectrometry was used to find proteins interacting with CKMT1, and Co-immunoprecipitation (Co-IP) and GST-pull down experiments were used to verify the interaction between proteins. The immunofluorescence (IF) assay was used to explore the functional position of CKMT1 in cells. The effect of CKMT1 expression level on the efficacy of paclitaxel (TAX) in the treatment of NSCLC was analyzed by a combined TAX experiment in vivo and in vitro.

**Results:**

CKMT1 expression was increased in NSCLC and CKMT1 promoted the proliferation of NSCLC cells in vitro and in vivo. CKMT1 knockdown resulted in a significantly increased G0/G1 fraction and decreased S phase cell fraction, indicating G1 phase arrest. Mechanically, the cyclin-dependent kinase 4 (CDK4) was identified to interact with CKMT1, and the crucial binding areas were focused on the DH domain of CKMT1 and the N- and C-terminal of CDK4. A fraction of the CDK4 proteins colocalize and interact with the CKMT1 at mitochondria, the level of phosphorylated CDK4 was regulated by CKMT1. Hence, the decrease in CKMT1 expression level could increase the antitumor effect of G2/M cell cycle antagonist-TAX in NSCLC in vitro and in vivo.

**Conclusions:**

CKMT1 could interact with CDK4 in mitochondria and regulate the phosphorylated level of CDK4, thus contributing to the proliferation and cell cycle transition of NSCLC cells. And CKMT1 could be a potential target to improve the sensitivity of chemotherapy based on TAX.

**Supplementary Information:**

The online version contains supplementary material available at 10.1186/s12931-023-02417-2.

## Introduction

Lung cancer is the most prevalent malignancy and the first leading cause of cancer deaths across the globe and accounting for approximately 18% of all cancer deaths in China [1, 2]. Non-small cell lung cancer (NSCLC), the major type of lung cancer, represents > 80% of all lung carcinoma cases [3]. Most patients diagnosed in the advanced stage, and carried a 5-year survival rate of ∼10–15% due to a lack of effective screening and therapy strategies [4–6]. Over the past decades, although advances in the understanding of the molecular pathogenesis and the genomics of lung cancer have led to the development of molecular subtyping and targeted therapy, it is extremely urgent to understand molecular mechanisms and identify key factors that drive lung cancer complex pathogenesis [7–9].

Kinases play key roles in tumorigenesis signal pathways of NSCLC and regulate a wide variety of cellular processes, including cell proliferation, differentiation, apoptosis, and metabolism [10–12]. Dysregulation of receptor tyrosine kinase (RTK) signaling leads to lung cancer, such as activating EGFR mutations are present in 15% of NSCLC, BRAF and KRAS always also mutated in lung cancer [13, 14]. Thus, targeting abnormal kinases is an important strategy in the treatment of tumors recently. However, for many kinases are involved in complex cellular processes, and acquired resistance inevitably occurs after several months of treatment, which results in poor patient outcomes, it is necessary to elucidate the novel regulatory mechanisms involved in NSCLC tumorigenesis.

Mitochondrial creatine kinase 1 (CKMT1/MtCK1) is a mitochondrial protein that exists on the outer surface of the inner membrane of mitochondria. CKMT1 is identified to facilitate the transfer of phosphocreatine (P-Cr) energy across mitochondria by transferring phosphate groups from mitochondrial ATP to creatine (Cr) [15]. CKMT1 is normally expressed at a high level in liver cancer, lung cancer, and breast cancer tissues. It was reported to promote the malignant growth of tumor cells, which is associated with a poor prognosis in patients [16–18]. In addition, CKMT1 can be a target to increase the efficacy of radiotherapy in nasopharyngeal carcinoma [19]. However, the potential role of CKMT1 in NSCLC remains unknown, thus investigating the molecular mechanism of CKMT1 needs to be implemented.

In this study, we identified that CKMT1 as a regulator of the cell cycle by targeting cyclin-dependent kinase 4 (CDK4) in NSCLC. Mechanistically, CKMT1 bound to CDK4 in mitochondrial, and then assisted in activation or nuclear translocation of CDK4, ultimately promoting the G1-S phase transition, and also rendered NSCLC resistant to G2/M cell cycle antagonist. Therefore, our results outline a novel mechanism for the control of CDK4 activity and suggest CKMT1 as a potential target for the therapy of NSCLC.

## Material and methods

### Cell lines

The human NSCLC cell lines A549, PC9, and H1299 were preserved in the State Key Laboratory of Oncology in South China, and cultured according to their guidelines. A549, PC9, H1299, and the corresponding modified cells were cultured at 37 °C in RPMI1640 (Gibco, USA) supplemented with 10% fetal bovine serum (Invitrogen, USA), 100 U/mL penicillin, and 100 μg/mL streptomycin in a humidified atmosphere containing 5% CO2. HEK-293 T cells were cultured in Dulbecco’s modified Eagle medium (Gibco, USA). All cell lines were authenticated by short tandem-repeat (STR) DNA profiling (Microread Diagnostics Co., Ltd, Guangzhou, China).

### Stable cell line construction

Complementary DNAs of CKMT1 and CDK4 were inserted into the pCDH-EF1-MCS-T2A-Puro vector with 3 × Flag at the N-terminus (System Bioscience, Palo Alto, CA, USA), and short hairpin RNAs (shRNAs) targeting CKMT1 (CKMT1-sh1: CGTGGAATTTGGCACAACAAT and CKMT1-sh2: CGGTGTCTTTGATATTTCTAA) and negative control shRNA (shNC: CAACAAGATGAAGAGCACCAA) were cloned into a pLKO.1 vector (Sigma-Aldrich, St. Louis, MO, USA). The doxycycline-inducible lung cancer cell lines were constructed using Tet-on inducible expression system following the manufacturer’s instructions. These plasmids were then packaged in 293 T cells to obtain recombinant lentiviruses with a Lentiviral Packaging Kit (FulenGen). Following a 48 h period of infection with lentivirus plus 5 mg/ml Polybrene, stably overexpression or knockdown cell lines were selected with 3 μg/mL puromycin for 3 days as previously described [20, 21].

### siRNA transfection

We transfected small interfering RNA-targeting candidate genes or Negative control (NC) siRNA (Sigma) into adherent cells using Lipofectamine 2000 or 3000 reagent (Invitrogen), according to the manufacturer’s guidelines. The sequences of each siRNA are listed in Additional file 2: Table S1.

### Generation of CRISPR/Cas9 KO cell lines

CKMT1 knockout cells were generated using the CRISPR-Cas9 system. The single-guide RNAs (sgRNAs) sequences targeting CKMT1 were designed from Zhang’s laboratory website (http://crispr.mit.edu/). The sgRNA sequences were sgRNA1: TACGAGCTGCCAGTGAACGA and sgRNA2: GGACCGACTAGGCAAATCAG. The sgRNAs were cloned into the Lenti-CRISPER v2 vector (Addgene#52961). Lentiviral particles were produced in 293 T cells. Positive clones were selected with 1 μg/ml puromycin and confirmed by DNA sequencing. The loss of CKMT1 protein expression was verified with the CKMT1 antibody by Western blot.

### Immunoprecipitation and Western blotting

Immunoprecipitation (IP) and Western blotting (WB) were performed as previously described [22–24]. Briefly, cultured cells were lysed in lysis buffer (CST, MA, USA) with PMSF, protease inhibitors (Roche, Basel, Switzerland) and phosphatase inhibitors (KeyGen Biotech, Nanjing, China). Protein concentrations were measured with a BCA protein assay kit (Beyotime, Haimen, China) according to the instruction. For IP [22], cell lysates were incubated with unconjugated primary antibodies at 4 °C overnight, followed by 4 h incubation with protein G agarose beads (Santa Cruz, CA, USA) at 4 °C. Then the precipitated protein complexes were washed four times with Tris-buffered saline (TBS) containing 0.05% Tween (TBS-T), boiled in 1 × loading buffer for 10 min, and then analyzed by WB. For WB [23, 24], protein samples were separated by sodium dodecyl sulfate–polyacrylamide gel electrophoresis (SDS-PAGE) and transferred to polyvinylidene fluoride (PVDF) membranes. After blocking nonspecific binding in 5% non-fat milk, the blots were incubated with specific primary antibodies against CKMT1, CDK4, p-CDK4, c-Myc, IgG, Flag, VDAC1, p-Rb, β-Actin, β-Tubulin, COX IV, GAPDH and GST, followed by reaction with HRP-conjugated antibodies (CST, MA, USA). All antibodies were diluted to 1: 1000 in the blotting. Signals were visualized using an ECL detection system (Amersham Biosciences).

### GST pull down

Purification of recombinant GST fusion proteins expressed in E. coli Agilent BL21 was transformed with pGEX4T1 or its derivatives containing the CKMT1, CDK4 coding sequences. GST pull-down reactions were conducted as follows [25]. Beads (50 μl) were coated with 10 μg GST fusion protein or GST control in PBS and incubated with specified amounts of soluble recombinant protein or cell lysates in a final volume of 0.5 ml of reaction buffer (50 mM HEPES, pH 7.4, 5 mM MgCl2, 150 mM NaCl, 0.1% Triton X-100, 0.1% bovine serum albumin (BSA) and 1 mM PMSF). The samples were incubated at 4 °C for 6 h or overnight while rotating. The beads were washed three times with PBS at 4 °C, boiled in 1 × loading buffer for 10 min, and then analyzed by SDS-PAGE.

### Immunofluorescence (IF) analysis

Cells in confocal dishes were fixed in 4% paraformaldehyde, permeabilized using 0.5% Triton X-100, and blocked with 3% BSA (Beyotime, Shanghai, China). After being blocked in BSA for 1 h, the cells were incubated with primary antibody against c-Myc or Flag or CKMT1 or CDK4 at 4 °C overnight. Next, the secondary antibody conjugated with Alexa Fluor 488 Dye (Invitrogen, CA, USA) or Alexa Fluor 594 was used to label the primary antibody for 1 h at room temperature in the dark. Then the samples were co-stained with DAPI (Beyotime, Shanghai, China). Photographs were captured and analyzed using a confocal laser scanning microscope.

### Gene expression array and GSEA analysis

The Dataset GSE19804 was obtained from NCBI Gene Expression Omnibus (GEO) (https://www.ncbi.nlm.nih.gov/geo/) and used to analyze expression differences of kinase genes in NSCLC and control lung tissues. Total RNAs were extracted from cultured CKMT1 knockout A549 cells and controls (or cells stably expressing shCKMT1 and control vector) using TRIZOL Reagent (Invitrogen, CA, USA) according to the manufacturer's instruction. The quantity and quality of total RNAs were measured and purified. Next, the total RNAs were subjected to Affymetrix PrimeView Human Gene Expression Array conducted by Boho Biotech Company, Shanghai, China. Differentially expressed genes (DEGs) between the CKMT1 knockout A549 cells and control cells were analyzed by gene set enrichment analysis (GSEA) software (Broad Institute, San Diego, USA) to find gene sets enriched by CKMT1 knockout or knockdown. Pathway enrichment analysis was performed using KEGG.

### Cell viability assays

Cell proliferation was assessed by CCK-8 Cell Counting Kit (Dojindo Laboratory, Kyushu, Japan) and colony formation assay. For the CCK-8 assay, cells were cultured into 96-well plates at a density of 2000 cells per well and incubated for 7 days. The growth rate of cells was determined by OD_450nm_ using CCK-8. For colony formation assay, cells (1000/well) were seeded into 6-well plates and cultured for 10–14 days, followed by staining with 0.5% crystal violet. Images of Colonies were captured and the number of colonies was counted by Image J software.

### Cell cycle assay

Cell cycle assay was performed by flow cytometry. Cells were fixed with ethanol 70% and incubated at − 20 °C for at least 24 h. Cells suspension was subsequently centrifuged and washed with ice-cold PBS, followed by staining in propidium iodide (PI) (20 μg/mL RNase, 50 μg/mL PI, and 0.1% (v/v) Triton X-100 in PBS) for 30 min at 37 °C water bath in the dark. The cell cycle stage was determined by flow cytometry and analyzed by FlowJo software.

### Animal model

Animal experiments were approved by the Sun Yat-sen University Cancer Center Institutional Animal Care and Usage Committee. Female BALB/c nude mice (4–5 weeks old, 15–18 g) were purchased from the SLRC laboratory Animal Co. (Shanghai, China). Mice were used to generate xenograft models via subcutaneous transplant of tumor sections (approximately 5 mm^3^) from lung cancer cell xenografts into the right flank. For Dox-dependent tumor experiments, mice were fed with Dox-containing sugar water 7–14 days after tumor transplantations. The volume and weight of tumors were monitored and evaluated. The tumor volume was calculated using the following formula [26]: V = 0.52 × length × width^2^.

### Statistical analyses

All experiments were repeated at least three times. The data were expressed as mean ± SD. All statistical analyses were performed using the GraphPad Prism 5.0 or SPSS version 22.0 statistical software (SPSS Inc, Chicago, IL, USA). The data obtained from in vitro and in vivo experiments of cell lines were assessed with Student’s 2-tailed t-tests. A value of *P* < 0.05 was considered a significant difference.

## Results

### Identification of CKMT1 as a contributor to proliferation in NSCLC cells

To identify potential NSCLC-associated kinase genes, we analyzed the expression differences of the top 50 kinase genes positively regulated in lung cancer compared to the normal control in microarray data from GEO datasets (GSE19804) (Additional file 3: Fig. S1A). 7 genes (CKMT1, HKDC1, AURKA, FRK, CHEK1, EIF2AK1, and BORA) were further evaluated as candidate genes involved in NSCLC tumorigenesis excluded those genes are well studied in cancer or the value of Log_2_FC < 1 (Additional file 3: Fig. S1B). To learn more about the function of these candidate genes, we performed a functional assay using small interfering RNAs (siRNAs) to knock down these genes in A549 cells respectively (Additional file 2: Table S1). The result showed that the decrease in cell proliferation is most significantly compared to the others in CKMT1-knockdown cells (Additional file 3: Fig. S1B). Additionally, we detected CKMT1 expression in NSCLC by immunohistochemistry (IHC) staining. The results showed that CKMT1 expression was markedly elevated at the protein level (68.6%; 129/188) in NSCLC tissues compared to paired normal tissues (P < 0.001; Additional file 3: Fig. S2A, B). These data suggest that CKMT1 is potentially involved in the regulation of NSCLC cell proliferation.

**Fig. 1:**
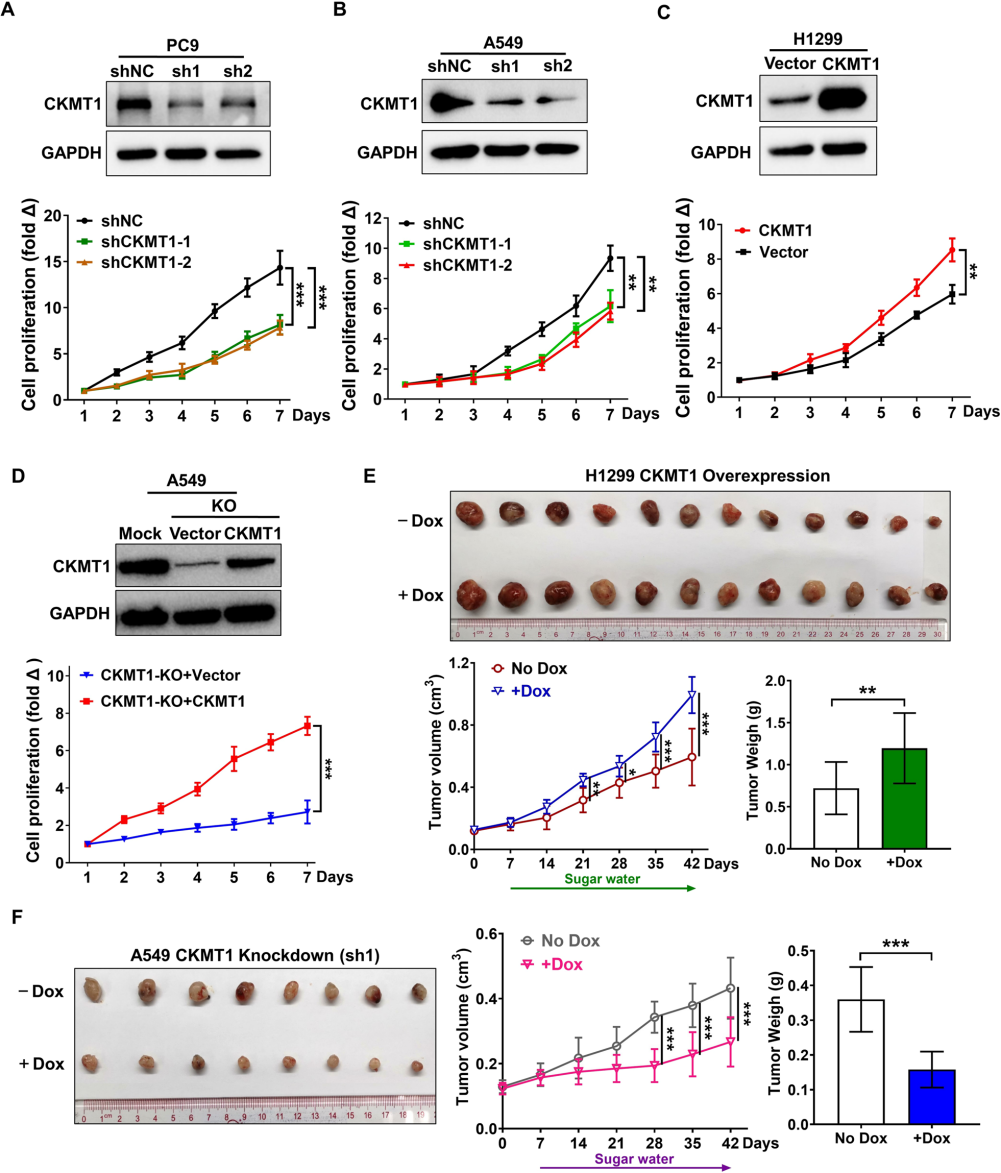
CKMT1 promotes the proliferation of NSCLC cells and the growth of NSCLC xenografts in nude mice. **A**–**C** The cell proliferation assays in PC9, A549 and H1299 cells by CCK8. **D** CKMT1 functional rescue assay in CKMT1-knockout (KO) A549 cells. **E** Tumor volume and tumor weight were measured in xenografts derived from induced CKMT1-overexpression H1299 cells. **F** Tumor volume and tumor weight were measured in Tet-on inducible sh-CKMT1 expression A549 models. Xenograft models were established via subcutaneous transplant of tumor sections derived from subcutaneous injection of doxycycline-inducible lung cancer cell lines expressing CKMT1 or shCKMT1. After solid tumors were established, the mice were administrated with sugar water containing Dox for inducing the corresponding CKMT1 or shCKMT1 expression. Blots and columns, mean; bars, standard deviation; *P < 0.05; **P < 0.01; ***P < 0.001

Next, we knocked down CKMT1 expression in PC9 or A549 cells respectively, and then overexpressed CKMT1 in H1299 cells and performed CCK8 assays to assess cell proliferation. The results showed that CKMT1 knockdown significantly suppressed the proliferation in PC9 and A549 cells, whereas ectopic overexpression of CKMT1 significantly enhanced the proliferation in H1299 cells (Fig. 1A–C). In addition, the ability of proliferation can be recovered when the CKMT1 was re-expressed in CKMT1-knockout A549 (A549 CKMT1-KO) cells through the functional rescue assay (Fig. 1D). Thus, we know that CKMT1 promotes the proliferation of lung cancer cells in vitro from the results mentioned above.

We then tested whether CKMT1 could influence the growth of NSCLC xenografts in vivo. We established the doxycycline-inducible CKMT1 overexpressed H1299 cells or knockdown A549 cells using Dox-on inducible expression system. Then we generated a xenograft model via subcutaneous transplant of tumor sections derived from subcutaneous injection of CKMT1 or shCKMT1-expression cultured cells. The results in nude mice showed that the volumes and weights of tumors generated from CKMT1-induced H1299 cells were significantly larger than those tumors without inducing CKMT1 overexpression in mice through feeding sugar water containing Dox (Fig. 1E). Consistent with the results obtained from the up-regulation experiments, the volumes and weights of tumors from the Dox group increased more slowly than those of the No Dox group that mice were fed without Dox containing sugar water (Fig. 1F). Collectively, these findings indicate that CKMT1 promotes cell proliferation in vitro and in vivo and contributes to NSCLC tumorigenesis.

### CKMT1 is essential for the G1-S phase transition

**Fig. 2:**
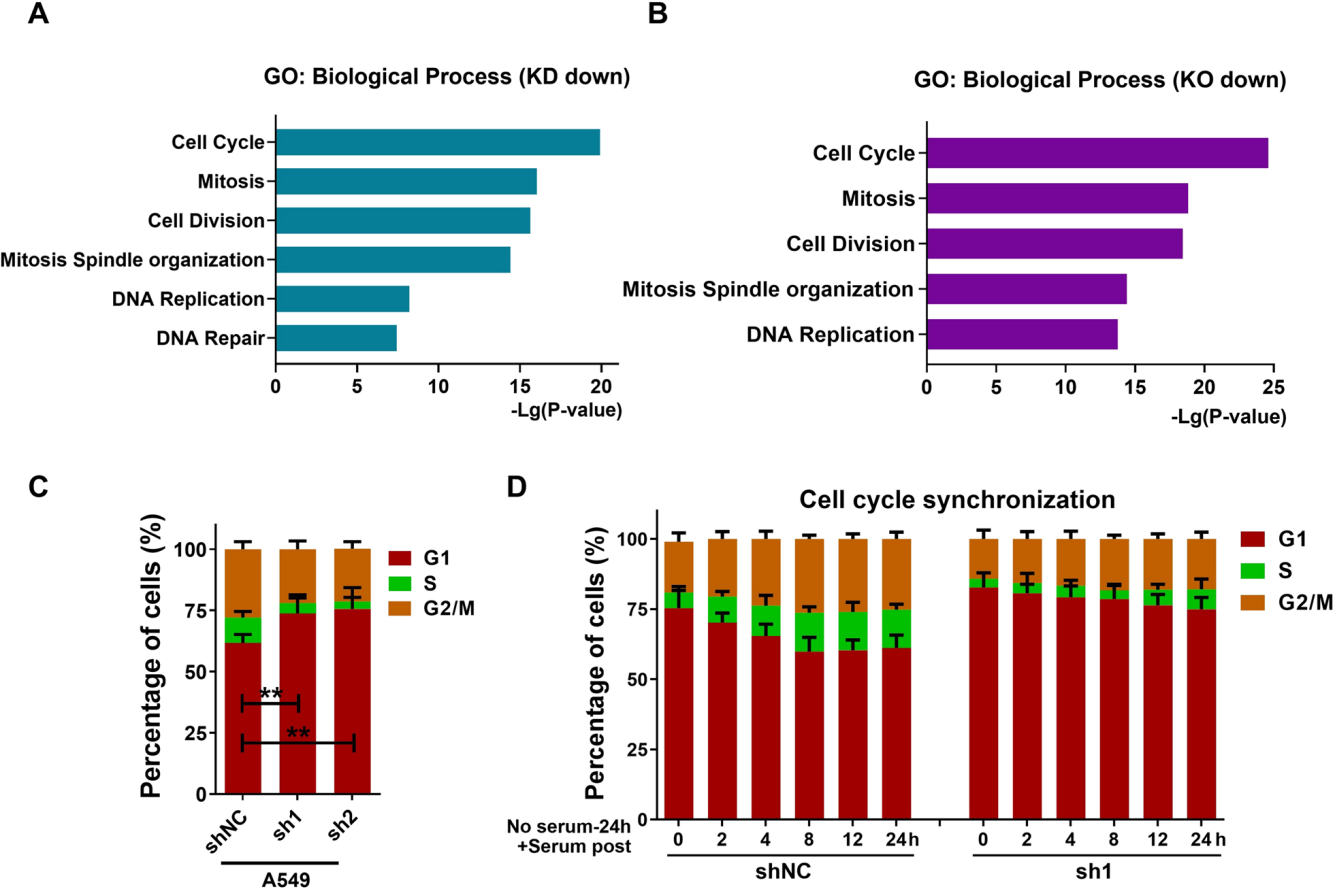
CKMT1 regulates the cell cycle of NSCLC cells and knockdown of CKMT1 renders cell cycle G1 phase arrest. **A** GO pathway analysis after CKMT1-knockdown (KD) in A549 cells. **B** GO pathway analysis after CKMT1-knockout (KO) in A549 cells. **C** The cell cycle distribution of A549-shNC and A549-shCKMT1 cells. **D** Results of cell cycle progression in A549-shNC and A549-shCKMT1 cells after 24 h of serum starvation. **P < 0.01

To further investigate the molecular mechanisms of CKMT1 on NSCLC tumorigenesis, we performed a genome-wide transcriptome analysis using RNA-Seq in CKMT1 knockdown or knockout A549 cells (Additional file 1). The results of GO enrichment analysis indicated that the cell cycle pathway was the most significantly enriched pathway associated with the down-regulation of CKMT1 (Fig. 2A, B). Then we performed a cell cycle assay by flow cytometry to confirm the result. Compared to control cells, CKMT1 knockdown resulted in a significantly increased G0/G1 fraction and decreased S phase cell fraction, indicating G1 phase arrest (Fig. 2C). On the contrary, the proportion of cells in the G1 phase was lower than the control group after overexpressing CKMT1 in H1299 cells, while the proportion of S phase cells increased significantly (Additional file 3: Fig. S2C). Additionally, we also performed a cell cycle analysis with synchronization using serum starvation to further elucidate the role of CKMT1 in modulating the cell cycle. Compared to the control cells, we found that CKMT1 deficient cells exhibited more resistance to G1 phase arrest induced by serum starvation (73.8–82.7% vs. 61.8–75.3%). After adding serum, CKMT1 deficient cells entered the S phase from G0/G1 phase more slowly, and the proportion of these cells was significantly lower than CKMT1 wide type cells (Fig. 2D). These data suggest that CKMT1 plays an important role in regulating the G1-S phase transition in NSCLC cells.

### CKMT1 interacts with CDK4

**Fig. 3:**
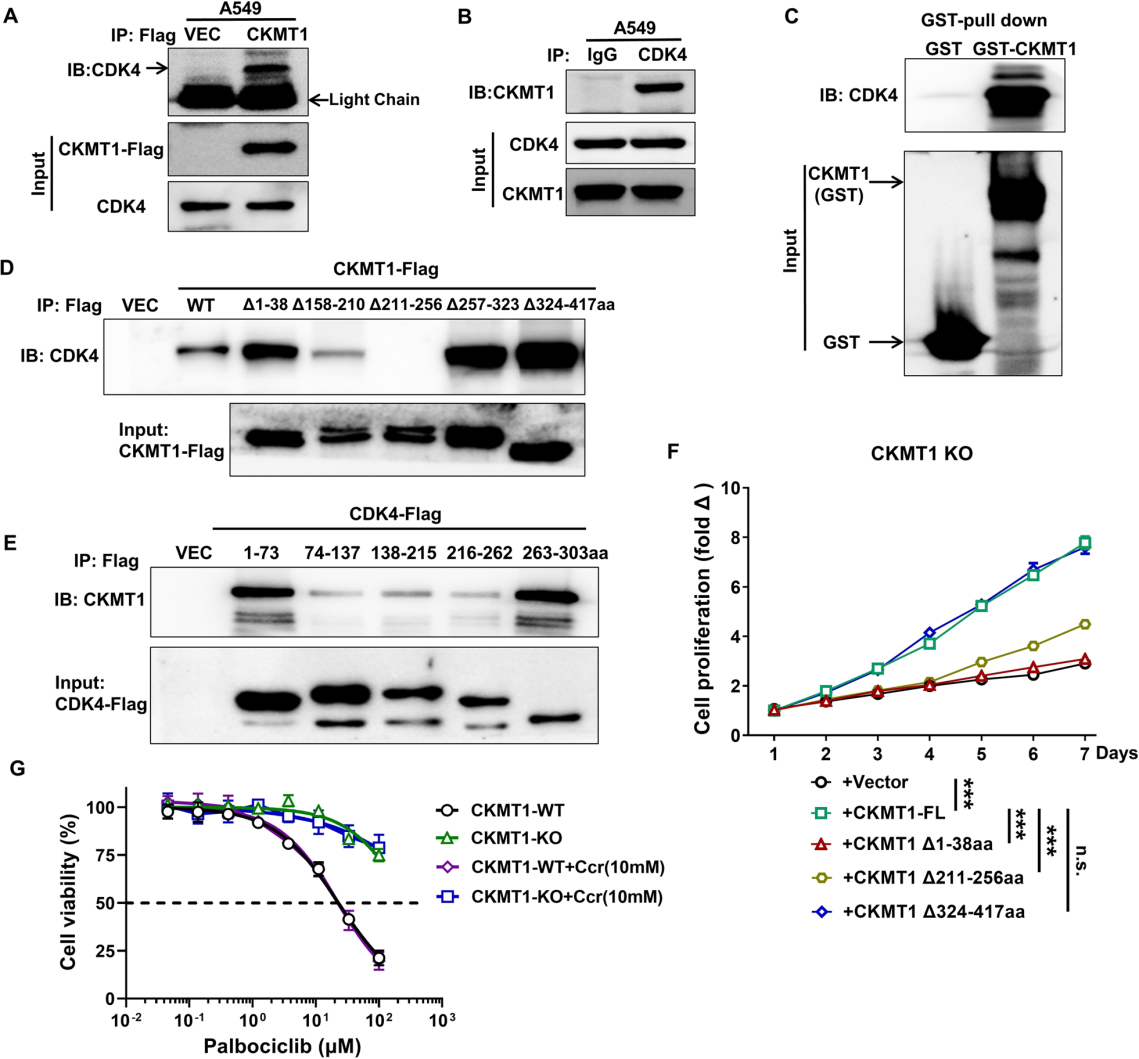
CKMT1 interacts with CDK4 in cells and the lysate. **A**, **B** Interaction of CKMT1 with CDK4 was determined using the Co-immunoprecipitation (co-IP) assay. **C** Interaction of CKMT1 with CDK4 was determined using the GST Pull-down assay. **D**, **E** Immunoblot analyses were performed with Flag-tagged truncated CKMT1 mutants (**D**) and truncated CDK4 mutants (**E**). **F** The cell proliferation assays of various deletions of CKMT1 in CKMT1-knockout A549 cells. **G** The cell viability assays in A549 cells treated with Palbociclib plus Cyclocreatine (Ccr). ***P < 0.001

To determine the molecular targets of CKMT1 in regulating the cell cycle, we performed a Co-IP assay for Mass Spectrometry (MS) analysis in A549 cells, and CDK4 was identified. CDK4 is a protein-serine kinase involved in the cell cycle, which interacts with cyclin D1 to form the cyclinD1-CDK4 complex, and then phosphorylates retinoblastoma (Rb) to release transcription factor E2F, thus promoting the cell from G1 phase to S phase [27]. To confirm the interactions between CKMT1 and CDK4, we performed co-IP assays and found that CKMT1 and CDK4 could interact with each other in A549 cells (Fig. 3A, B). In addition, the results of the GST pull-down assay showed that CDK4 could directly interact with purified GST-CKMT1 (Fig. 3C).

To further determine the binding fragment of CKMT1 with CDK4, we generated a series of plasmids containing Flag-tagged different truncated fragments of CKMT1 and CDK4 (Fig. 3D, E) and investigated the effects of these deletions or truncations on binding with each other in A549 cells. As known in Fig. 3D, the binding of CKMT1 with CDK4 was completely abolished by the removal of the fragment of 211–256aa, indicating that the Dbl homology domain 2 (DH2) was required for the binding of CKMT1 to CDK4, rather than the domain of creatine phosphorylation (CPS, the fragment of 257–323aa). For CDK4, we further found that the truncated N- and C-terminal variants of CDK4 could interact with CKMT1 (Fig. 3E). These results support a direct interaction between CDK4 and CKMT1, and the crucial binding areas were focused on the DH domain of CKMT1 and the N- and C-terminal of CDK4.

To investigate whether the function of CKMT1 in the cell cycle is related to its binding ability to CDK4, we performed CCK8 assays to assess cell proliferation of various deletions of CKMT1 in CKMT1-knockout A549 cells. The results showed that the ability of proliferation can be completely recovered when the C-terminal fragment deleted CKMT1 (Δ324–417aa) was re-expressed; however, less recovery was observed when the DH2 deleted CKMT1 (Δ211–256aa) was re-expressed (Fig. 3F), these data suggest the function of CKMT1 is partly dependent on the interaction with CDK4. This suggestion had been verified further by another experiment, we found that CKMT1-knockout rendered A549 cells more resistant to CDK4 inhibitor-Palbociclib as compared with CKMT1 wide type cells, which could not be affected by creatine kinase inhibitor-Cyclocreatine (Ccr) (Fig. 3G). Together, these results demonstrate that CKMT1 functions by interacting directly with CDK4, and this process was unrelated to the activity of creatine phosphorylation.

### CKMT1 regulates the activation of CDK4 in mitochondria

**Fig. 4:**
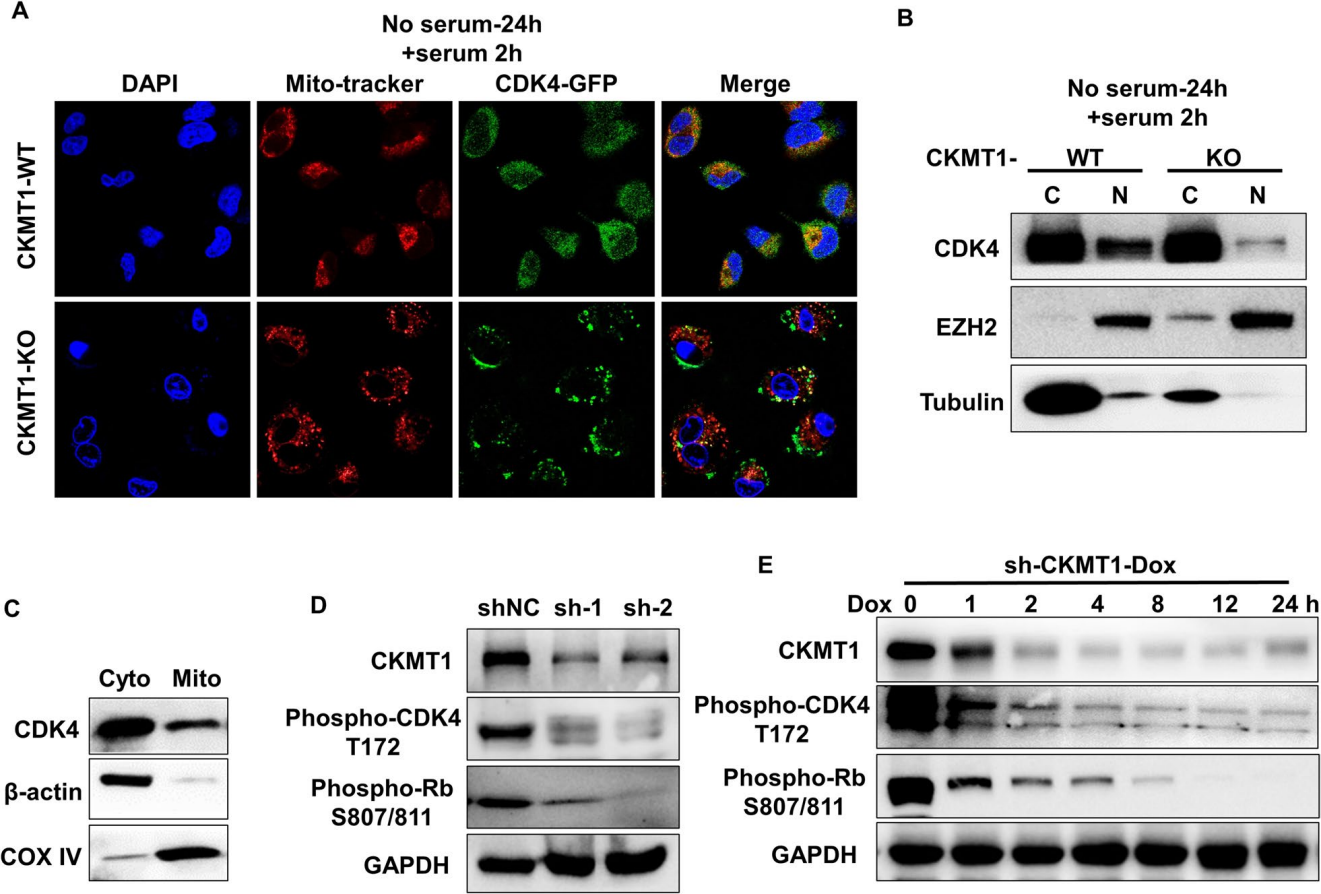
CDK4 is partly located in mitochondria and CKMT1 could regulate the phosphorylation of CDK4. **A** Immunofluorescent staining of mitochondrion tracker (red) and CDK4 (green) using confocal microscopy in A549 cells. **B** The western blot analysis of CDK4 in cytosolic (C) and nuclear (N) fractions of A549 cells. **C** The western blot analysis of CDK4 in mitochondrial and cytosolic fractions of A549 cells. **D**, **E** Immunoblot analyses were performed with CKMT1, p-CDK4 and p-Rb through in A549 cells. cyto: cytosolic fractions; mito: mitochondrial fractions

Since the recovery ability was completely abolished by the removal of mitochondrial presequence (Δ1-38aa) in CKMT1-KO cells (Fig. 3F), which means CKMT1 only exhibited the function of regulating proliferation in mitochondria, we then asked whether CDK4 may enter to mitochondria for interaction with CKMT1. For this, we carried out living cell imaging to assess. The result of confocal colocalization studies demonstrated that CDK4 (GFP) is partly located in mitochondria when added serum 2 h after synchronization in CKMT1-WT cells, however, knockout of CKMT1 abrogated localization of CDK4 to mitochondria (Fig. 4A), these means that CKMT1 plays a key role in rendering CDK4 located in mitochondria. To further confirm this, we performed mitochondrial isolation for western blot, to identify CDK4 could present in mitochondrial fractions (Fig. 4C). Of note, we also observed that the deficiency of CKMT1 markedly suppressed the nuclear translocation of CDK4 (Fig. 4B). Taken together, these results indicate that a fraction of the CDK4 proteins colocalize and interact with the CKMT1 at mitochondria, thus influencing the transition of the cell cycle.

CDK4, a cyclin-dependent kinase, is well known to phosphorylate Rb to promote G1/S cell cycle transition, and the activity of kinase is determined by its phosphorylation [28]. To better understand the regulation of CKMT1 on the activation of CDK4, we detected the phosphorylated level of CDK4 and its downstream Rb. As expected, we found the level of phosphorylated CDK4 and phosphorylated Rb decreased when we knocked down CKMT1 expression in A549 cells (Fig. 4D). In addition, Dox-induced shCKMT1-expression resulted in gradually decreased levels of phosphorylated CDK4, as well as the levels of phosphorylated Rb (Fig. 4E). These results suggest a potential role for CKMT1 in ultimately regulating the cell cycle via phosphorylation of CDK4 to activate downstream transcriptional responses.

### Knockdown of CKMT1 enhances the antitumor effect of paclitaxel

**Fig. 5:**
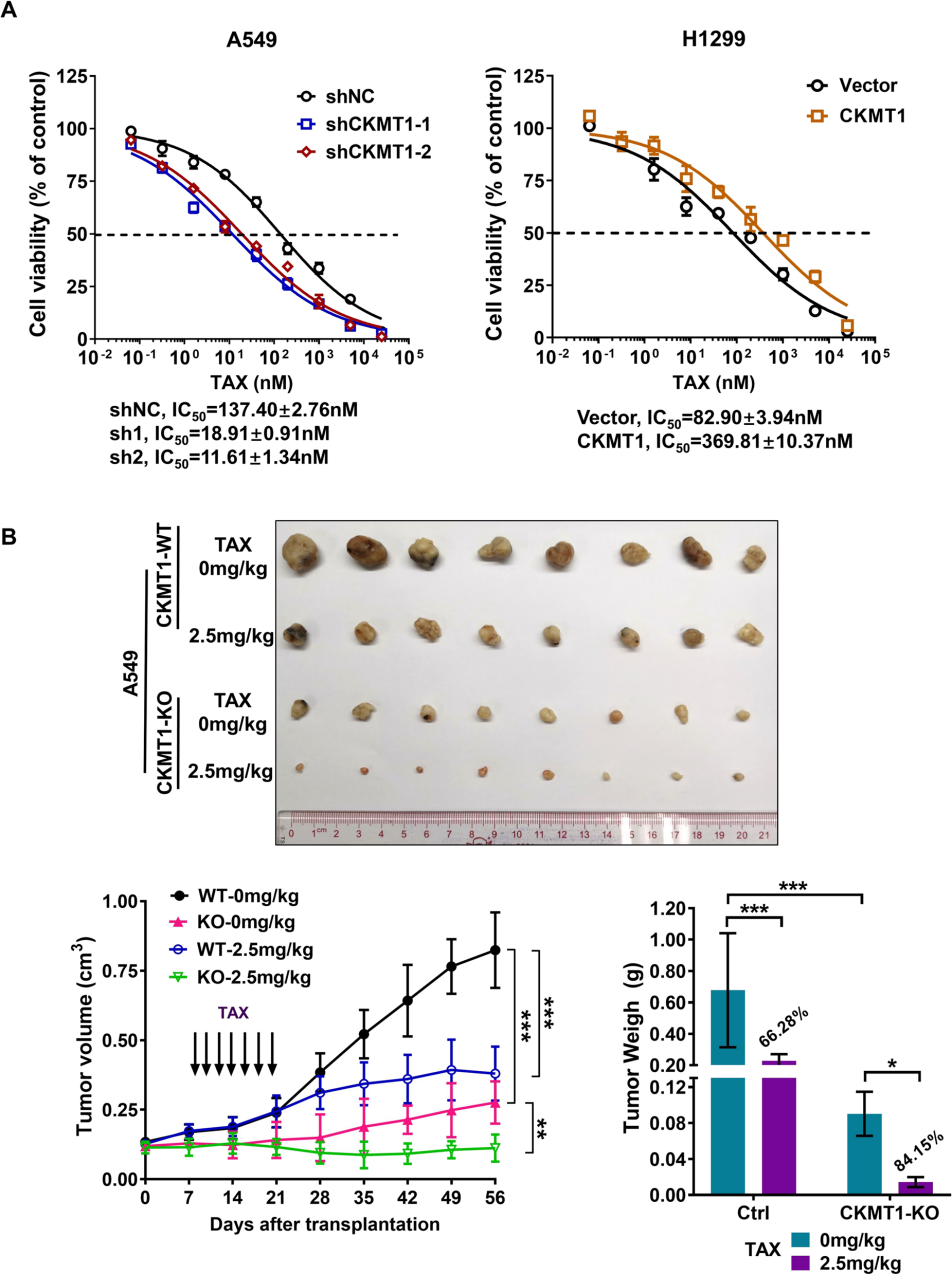
Down-regulation of CKMT1 enhances the antitumor effect of paclitaxel (Tax). **A** Cell viability curves and IC_50_ values of Tax for A549 and H1299 cells. **B** Tumor volume and tumor weight was measured in xenografts derived from CKMT1-knockout (KO) A549 cells in the combination of Tax treatment. Blots and columns, mean; bars, standard deviation; *P < 0.05; **P < 0.01; ***P < 0.001

Paclitaxel (TAX) as a tubulin inhibitor, has been used to inhibit cancer cell growth and induce G2/M cell cycle arrest [29, 30]. Thus, we asked whether tumor cells with lower CKMT1 expression might have increased sensitivity to TAX. To test this possibility, we added the G2/M cell cycle arrestor TAX to A549 control and CKMT1 knockdown cells in vitro and in vivo. Our results showed that knockdown of CKMT1 did significantly decrease the IC_50_ of paclitaxel compared to A549 shNC cells (IC_50_ = 137.40 ± 2.76 nM in A549 shNC vs. IC_50_ = 18.91 ± 0.91 nM in A549 shCKMT1-1 and 11.61 ± 1.34 nM in A549 shCKMT1-2, P < 0.01). Meanwhile, CKMT1 overexpressed cells (IC_50_ = 369.81 ± 10.37 nM) were 4.5-fold more resistive to paclitaxel than control vector cells (IC_50_ = 82.90 ± 3.94 nM) (Fig. 5A). In animal experiments, the tumor volume and tumor weight in the combination of CKMT1-KO and TAX treatment group were significantly reduced after TAX treatment compared to the control group (Figs. 5B). Therefore, CKMT1 affects the anti-tumor effect of paclitaxel and may be a potential target to improve the sensitivity to chemotherapy.

## Discussion

CKMT1, a key enzyme in mitochondrial energy metabolism, plays an important role in high energy requirements cells such as skeletal muscle cells, brain cells, and tumor cells [15, 18]. Cancer is a metabolic disease, and the function of CKMT1 in mitochondria plays an important role in the energy demand transformation process. CKMT1 helps maintain energy conversion and protects cells from death by preventing stressful situations such as hypoxia [31]. CKMT1 is always associated with malignancy in a variety of cancers, not only because previous studies have proved that it affects the proliferation and migration of cancer cells, but also CKMT1 is involved in the process by which tumor cells adapt to metabolic needs to grow fast in the presence of limited oxygen and glucose. However, CKMT1 seems to have different effects on the biological function of different types of tumor cells. According to previous reports, CKMT1 expression levels are increased in liver cancer, lung cancer, stomach cancer and breast cancer cells, and are related to the poor prognosis of patients [16, 17, 32, 33]. However, in oral cancer, prostate cancer and glioma, the expression level of CKMT1 is lower in tumor tissues than that in normal tissues [34, 35], and the low level of CKMT1 implied a poor prognosis in head and neck squamous cell carcinoma [36]. CKMT1 exerts different functions because cancer cells have different genetic backgrounds, which behavior is regulated by multiple factors and the combined effect of the microenvironment [18, 34].

In our study, we found that CKMT1 promoted NSCLC cell proliferation in vitro and in vivo. This finding is consistent with that of Ming Li et al. who also found knockdown of CKMT1 inhibited the proliferation, colony formation, invasion and EMT of H1650 and H1299 cells [18]. Moreover, our study demonstrated that CKMT1 influenced the cell cycle might through the interaction with protein CDK4, a most important regulator of the cell cycle. This is not reported in the research about CKMT1 at present. As we all know, cell proliferation was closely associated with the cell cycle and many proteins involved in these two processes are the main targets for the treatment of cancer. In the acute myeloid leukemia (AML) model, EVI1 promotes CKMT1 expression by repressing the myeloid differentiation regulator RUNX1. The inhibition of CKMT1 decreased the viability, promoted the cell cycle arrest and apoptosis of EVI1-positive cell lines, and prolonged survival in mouse models [37]. In addition, knocking out CKMT1 decreased STAT3 phosphorylation, thus increasing the radiosensitivity in Nasopharyngeal carcinoma therapy [19]. Combining our results in this study, we believe that CKMT1 can be an effective target for the treatment of lung cancer.

We give more evidence that part of CDK4 could locate in mitochondria. Additionally, there was also a study showed that Cyclin D1/CDK4 relocated to mitochondria and directly phosphorylates Manganese superoxide dismutase (MnSOD) to enhance its enzymatic activity and mitochondrial respiration for adaptive radioprotection in mammalian cells [38]. The findings can contribute to a better understanding of the physiological function of CDK4 in mitochondria. According to our results, CDK4 was colocalized with CKMT1 in mitochondria of lung cancer cells and its phosphorylation level is also regulated by CKMT1. The activity of CDK4 depended on its phosphorylation, then it phosphorylates RB and promotes G1/S cell cycle transition once it is activated. We found CKMT1 interacted with CDK4 through its DH domain, which was a catalytic center for the exchange reaction. CKMT1 plays the main function in energy metabolism—it maintains an energy balance between energy supply sites and demand sites using the easily diffusible creatine [39]. Therefore, the function of CKMT1 to phosphorylate CDK4 is a function different from creatine kinase, and it is important for cancer metabolism. CKMT1 might also alter cancer metabolism and energy generation through phosphorylating and interacting with CDK4 in the developing process of lung carcinogenesis.

Our results showed that CKMT1 affects the anti-tumor effect of paclitaxel and may be a potential target to improve the sensitivity to chemotherapy. Paclitaxel as a tubulin inhibitor, has been used to inhibit cancer cell growth and induce G2/M cell cycle arrest. According to our results, CKMT1 plays an important role in regulating the G1-S phase transition. We speculated whether reducing CKMT1 expression in NSCLC cells would increase cancer cells' sensitivity to chemotherapy with paclitaxel and our experiments demonstrated this. But in fact, we just found this synergy phenomenon, and it is not clear how the cell cycle changes when the combination of CKMT1 knockdown and TAX treatment. The mechanism of lower CKMT1 expression increased the anti-tumor effect of paclitaxel in NSCLC is not yet clear and we look forward to more research in the future to uncover it.

Despite the current study highlighting the importance of CKMT1 and its interaction with CDK4 in lung tumorigenesis, there are still several limitations that need to be pinpointed. Firstly, CKMT1 is a mitochondrial protein and part of CDK4 interacts with it in mitochondria, CDK4 regulates the cell cycle mostly in the nucleus. How does CKMT1 phosphorylated CDK4 enter the nucleus from the mitochondria? We didn't figure out the exact mechanism in this study. What’s more, the effects of CKMT1 on mitochondrial function and metabolism in lung cancer also need to be further studied.

## Conclusions

Taken together, CKMT1 promotes the proliferation and cell cycle progression from the G1 to the S phase of NSCLC cells in vitro and the growth of NSCLC xenografts in nude mice. These effects might depend on the interaction between CKMT1 and CDK4 in mitochondria and the phosphorylated level of CDK4 regulated by CKMT1, which contribute to the development and progression of NSCLC. Moreover, CKMT1 can be a target to improve the sensitivity of paclitaxel chemotherapy as it can regulate the cell cycle.

## Supplementary Information

**Additional file 1.** The genome-wide transcriptome analysis using RNA-Seq in CKMT1 knockout or knockdown A549 cells. A549KO, CKMT1 knockout A549 cells; A549KD, CKMT1 knockdown A549 cells.

**Additional file 2: Table S1.** The target sequences of siRNAs.

**Additional file 3: Figure S1.** (A) The heatmap of the expression differences of top 50 kinase genes positively regulated in lung cancer in GSE 19804. (B) Cell proliferation assay performed by CCK-8 assay for 7 candidate genes. **Figure S2**. (A) The heatmap of the CKMT1 protein expression in NSCLC tissues and paired normal tissues. (B) The comparison of the CKMT1 protein expression between NSCLC tissues and paired normal tissues. (C) The cell cycle distribution of H1299-ctrl and H1299-CKMT1 cells. *P<0.05; **P<0.01; ***P<0.001.

## Data Availability

Our experimental datasets used during the current study are available from the corresponding authors upon reasonable request.

## Abbreviations

NSCLC: Non-small cell lung cancer
CKMT1: Mitochondrial creatine kinase 1
P-Cr: Phosphocreatine
Cr: Creatine
CDK4: Cyclin-dependent kinase 4
STR: Short tandem-repeat
shRNAs: Short hairpin RNAs
sgRNAs: Single-guide RNAs
IP: Immunoprecipitation
WB: Western blotting
GEO: Gene Expression Omnibus
DEGs: Differentially expressed genes
GSEA: Gene set enrichment analysis
IHC: Immunohistochemistry
MS: Mass Spectrometry
Rb: Retinoblastoma
Ccr: Cyclocreatine
TAX: Paclitaxel
AML: Acute myeloid leukemia
MnSOD: Manganese superoxide dismutase
