# Supplementary material for: Mitochondrial creatine kinase 1 regulates the cell cycle in non-small cell lung cancer via activation of cyclin-dependent kinase 4
Source: Respir Res. 2023 Apr 15;24:111. doi: 10.1186/s12931-023-02417-2 (PMC10105958; doi:10.1186/s12931-023-02417-2)
Supplement: Supplementary file 2 — Additional file 2: Table S1. The target sequences of siRNAs. [file 12931_2023_2417_MOESM2_ESM.docx]

**Table S1.** **The target sequences of siRNAs**

| **Name** | **Target genes** | **Target sequence** |
| --- | --- | --- |
| si-CKMT1 | CKMT1 | 5'- CGUGGAAUUUGGCACAACAAU-3' |
| si-HKDC1 | HKDC1 | 5'- CCUUGCUAAUACAAGAGAGAU -3' |
| si-AURKA | AURKA | 5'- ACGAGAAUUGUGCUACUUAUA -3' |
| si-FRK | FRK | 5'- GCUCCAUUUGAUUUGUCGUAU -3' |
| si-CHEK1 | CHEK1 | 5'- GUGACAGCUGUCAGGAGUAUU -3' |
| si-EIF2AK1 | EIF2AK1 | 5'- GCAGAAGUUCUAACAGGUUUA -3' |
| si-BORA | BORA | 5'- GCUGAUGAAUUUGCAGAUCAA -3' |
