# Supplementary material for: Mitochondrial creatine kinase 1 regulates the cell cycle in non-small cell lung cancer via activation of cyclin-dependent kinase 4
Source: Respir Res. 2023 Apr 15;24:111. doi: 10.1186/s12931-023-02417-2 (PMC10105958; doi:10.1186/s12931-023-02417-2)
Supplement: Supplementary file 3 — Additional file 3: Figure S1. (A) The heatmap of the expression differences of top 50 kinase genes positively regulated in lung cancer in GSE 19804. (B) Cell proliferation assay performed by CCK-8 assay for 7 candidate genes. Figure S2. (A) The heatmap of the CKMT1 protein expression in NSCLC tissues and paired normal tissues. (B) The comparison of the CKMT1 protein expression between NSCLC tissues and paired normal tissues. (C) The cell cycle distribution of H1299-ctrl and H1299-CKMT1 cells. *P<0.05; **P<0.01; ***P<0.001. [file 12931_2023_2417_MOESM3_ESM.pdf]

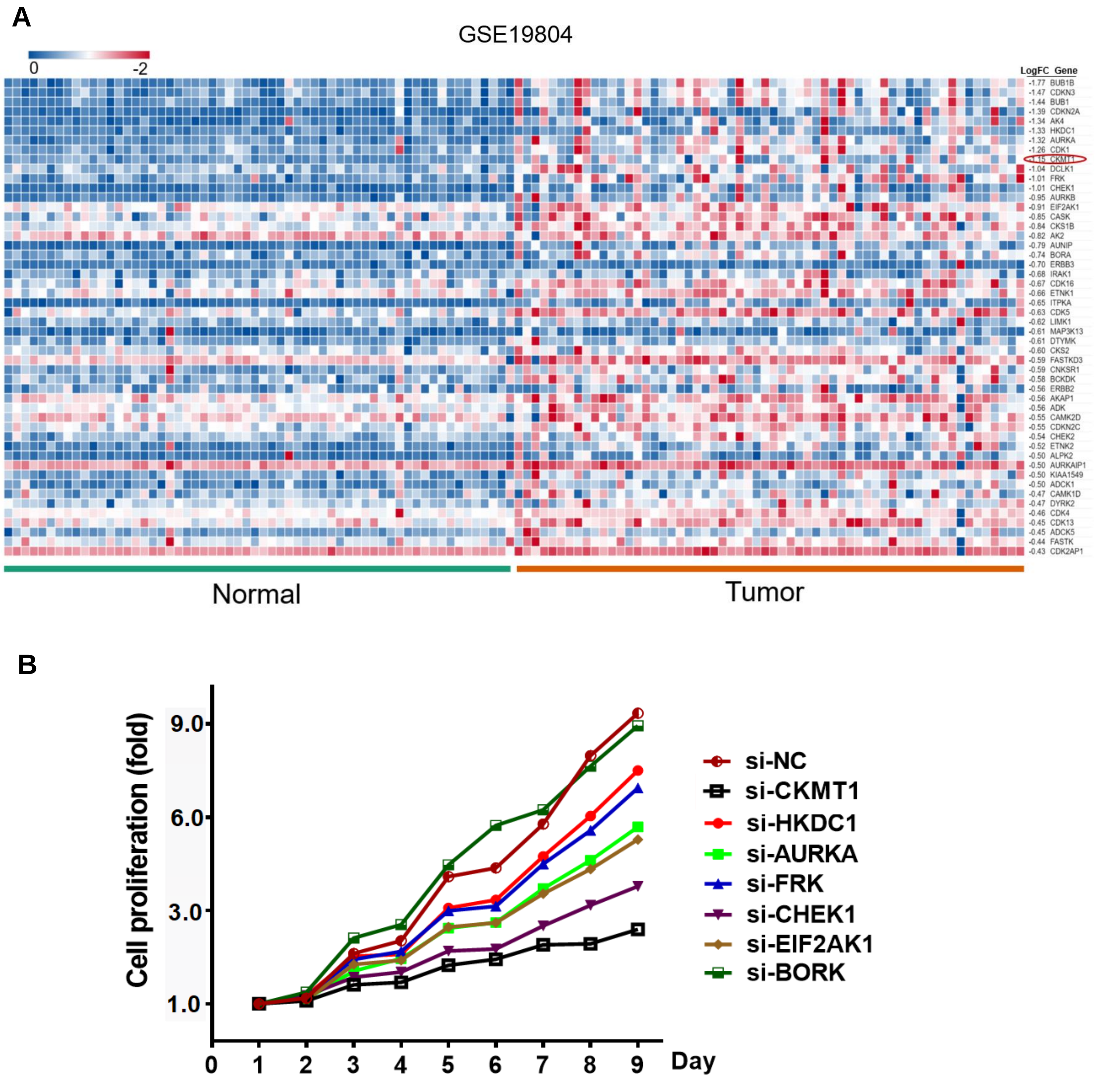

Figure S1. (A) The heatmap of the expression differences of top 50 kinase genes in GSE19804. (B)Cell proliferation assay performed by CCK-8 assay for 7 candidate genes.

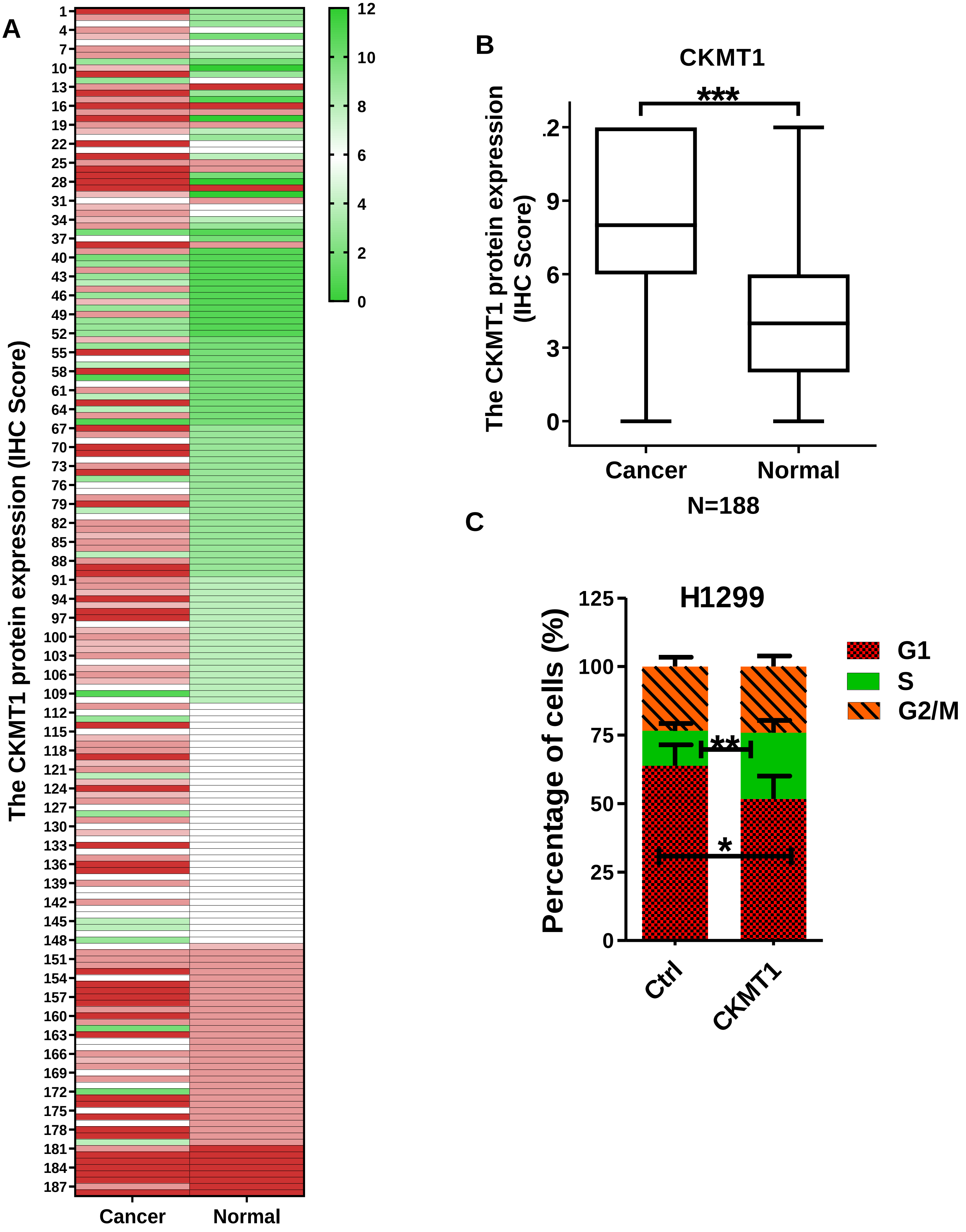

Figure S2. (A) The heatmap of the CKMT1 protein expression in NSCLC tissues and paired normal tissues. (B) The comparison of the CKMT1 protein expression between NSCLC tissues and paired normal tissues. (C) The cell cycle distribution of H1299-ctrl and H1299-CKMT1 cells. *P<0.05; **P<0.01; ***P<0.001.
